# Soluble Guanylate Cyclase α_1_–Deficient Mice: A Novel Murine Model for Primary Open Angle Glaucoma

**DOI:** 10.1371/journal.pone.0060156

**Published:** 2013-03-20

**Authors:** Emmanuel S. Buys, Yu-Chieh Ko, Clemens Alt, Sarah R. Hayton, Alexander Jones, Laurel T. Tainsh, Ruiyi Ren, Andrea Giani, Maeva Clerté, Emma Abernathy, Robert E. T. Tainsh, Dong-Jin Oh, Rajeev Malhotra, Pankaj Arora, Nadine de Waard, Binglan Yu, Raphael Turcotte, Daniel Nathan, Marielle Scherrer-Crosbie, Stephanie J. Loomis, Jae H. Kang, Charles P. Lin, Haiyan Gong, Douglas J. Rhee, Peter Brouckaert, Janey L. Wiggs, Meredith S. Gregory, Louis R. Pasquale, Kenneth D. Bloch, Bruce R. Ksander

**Affiliations:** 1 Anesthesia Center for Critical Care Research, Department of Anesthesia, Critical Care, and Pain Medicine, Massachusetts General Hospital, Harvard Medical School, Boston, Massachusetts, United States of America; 2 Department of Ophthalmology, Schepens Eye Research Institute, Harvard Medical School, Boston, Massachusetts, United States of America; 3 Department of Ophthalmology, School of Medicine, National Yang-Ming University, Taipei, Taiwan; 4 Wellman Center for Photomedicine and Center for Systems Biology, Massachusetts General Hospital, Harvard Medical School, Boston, Massachusetts, United States of America; 5 Department of Ophthalmology, Boston University School of Medicine, Boston, Massachusetts, United States of America; 6 Department of Ophthalmology, Massachusetts Eye and Ear Infirmary, Harvard Medical School, Boston, Massachusetts, United States of America; 7 Cardiology Division, Department of Medicine, Massachusetts General Hospital, Harvard Medical School, Boston, Massachusetts, United States of America; 8 Department of Biomedical Engineering, Boston University, Boston, Massachusetts, United States of America; 9 Channing Division of Network Medicine, Brigham and Women's Hospital, Harvard Medical School, Boston, Massachusetts, United States of America; 10 VIB Department of Molecular Biomedical Research, Ghent University, Ghent, Belgium; University of Rochester, United States of America

## Abstract

Primary open angle glaucoma (POAG) is a leading cause of blindness worldwide. The molecular signaling involved in the pathogenesis of POAG remains unknown. Here, we report that mice lacking the α_1_ subunit of the nitric oxide receptor soluble guanylate cyclase represent a novel and translatable animal model of POAG, characterized by thinning of the retinal nerve fiber layer and loss of optic nerve axons in the context of an open iridocorneal angle. The optic neuropathy associated with soluble guanylate cyclase α_1_–deficiency was accompanied by modestly increased intraocular pressure and retinal vascular dysfunction. Moreover, data from a candidate gene association study suggests that a variant in the locus containing the genes encoding for the α_1_ and β_1_ subunits of soluble guanylate cyclase is associated with POAG in patients presenting with initial paracentral vision loss, a disease subtype thought to be associated with vascular dysregulation. These findings provide new insights into the pathogenesis and genetics of POAG and suggest new therapeutic strategies for POAG.

## Introduction

Glaucoma is a progressive eye disease that ultimately leads to blindness due to the irreversible loss of retinal ganglion cells (RGCs) with concomitant optic nerve degeneration [1]. Over 4 million Americans and 65 million people worldwide have glaucoma, making it the leading cause of blindness in the US and the second leading cause of blindness worldwide. Although available therapies delay disease progression [1], protection remains incomplete and vision loss due to glaucoma cannot be regained, highlighting the need for novel therapeutic approaches and drug targets [2]. In primary open-angle glaucoma (POAG), one of the most common glaucoma subtypes, there is variable elevation of intraocular pressure (IOP) associated with impaired aqueous outflow that occurs despite normal anterior segment anatomy and an open iridocorneal angle [1].

Although multiple POAG risk factors have been identified [2], the etiology of POAG remains to be elucidated, likely because the disease can be stratified into various subtypes defined by discrete but yet unknown biochemical pathways. Two major pathophysiologic mechanisms for POAG have been proposed. In the “mechanical theory” [1], [2], [3], [4] optic neuropathy is caused by increased IOP, an important risk factor for glaucoma [5], [6]. While elevated IOP is currently the only risk factor amenable to treatment, some patients with high IOP do not develop POAG and other patients with low or normal IOP do, suggesting that other pathologies may contribute to the etiology of POAG [2], [6]. Alternatively, a vascular component has been hypothesized to contribute to POAG pathophysiology. Intravenous administration of the endothelial and NO-dependent vasodilator acetylcholine, fails to mediate brachial artery vasodilation in untreated POAG [7]. Also, flow-mediated vasodilation [8] and retinal vascular autoregulation [9] are impaired in POAG. Furthermore, POAG patients with initial paracentral visual field loss tend to have more frequent systemic vascular risk factors such as migraines and hypotension [10], and low ocular perfusion pressure (mean arterial blood pressure – IOP) is a risk factor for POAG [11], [12]. However, the extent to which vascular dysfunction contributes to glaucomatous optic neuropathy remains to be elucidated and is controversial [3], [4].

Nitric oxide (NO) is an attractive candidate as a factor that could modify both mechanical and vascular events in POAG pathogenesis. NO, an important modulator of smooth muscle function, is synthesized by a family of three enzymes referred to as NO synthases (NOSs), all of which are expressed in the eye [13], [14], [15]. NO activates the cGMP-generating heterodimeric enzyme soluble guanylate cyclase (sGC). sGC consists of one α and one β subunit and mediates many of the physiological effects of NO, including the ability of NO to relax smooth muscle cells [16], [17]. Two isoforms of each sGC subunit have been identified (α_1_, α_2_, β_1_, and β_2_), but only the sGCα_1_β_1_ and sGCα_2_β_1_ heterodimers appear to function in vivo [18].

NO-cGMP signaling has been suggested to participate in the regulation of aqueous humor (AqH) outflow and IOP [19], [20]. Preclinical studies have demonstrated the ability of NO-donor compounds to lower IOP [21], [22] and enhance tissue oxygenation of the optic nerve head [23]. Importantly, NO metabolites and cGMP levels are decreased in plasma and AqH samples from POAG patients [24], [25]. Moreover, two independent studies have identified NOS3 gene variants that are associated with POAG in women [26], [27]. A third study that did not find an association between NOS3 variants and POAG had a small sample size and did not provide gender specific results [28]. Together, these findings suggest that impaired NO-cGMP signaling can contribute to the etiology of POAG [29], [30]. Several mechanisms, including genetic variation and oxidative stress can regulate NO-cGMP signaling. However, the mechanisms by which NO-cGMP signaling modulates POAG risk and whether impaired NO-cGMP signaling can result in POAG remain unclear.

Here, we identify mice deficient in sGCα_1_ (sGCα_1_^−/−^ mice) as a new murine model of POAG characterized by age-related optic neuropathy, an age-related increase in IOP, and retinal vascular dysfunction. Moreover, in a nested case-control study, we identified a genetic association between the locus containing the genes encoding the α_1_ and β_1_ subunits of sGC and a subtype of POAG characterized by paracentral vision loss and vascular dysregulation.

## Materials and Methods

### Ethics statement

This study was carried out in strict accordance with the recommendations in the Guide for the Care and Use of Laboratory Animals of the National Institutes of Health. Housing and all procedures involving experimental animals (mice) described in this study were specifically approved by the Institutional Animal Care and Use Committees (IACUC) of Massachusetts General Hospital (Subcommittee on Research Animal Care), and the Schepens Eye Research Institute. All procedures were performed under adequate anesthesia, and all efforts were made to minimize suffering. The Institutional Review Board of the Massachusetts Eye and Ear Infirmary, Harvard School of Public Health, and Brigham and Women's Hospital approved the gene association studies described. All participants provided written informed consent. The Human Research Committees of Massachusetts Eye and Ear Infirmary, Partners Healthcare System, and the Harvard School of Public Health approved the consent procedure.

### Animals

Age-matched, one- to 17-month-old female sGCα_1_^−/−^ mice and WT mice on a 129S6 background were studied [31]. Mice were anesthetized (for AqH outflow measurements and SD-OCT) by intraperitoneal (IP) injection of ketamine (100 mg/kg) and xylazine (9 mg/kg) or (for IOP measurements and SLO experiments) with isoflurane (2%). Mice were euthanized (for tissue harvest) using pentobarbital (10 mg IP). Observers masked as to animal genotype performed all data acquisition and analyses described.

### Histology and immunohistochemistry

Human eyes (obtained from the New England Eye Bank (Newton, MA)) and mouse eyes were fixed in paraformaldehyde, embedded in paraffin, sectioned and incubated with antibodies specific for sGCα1, sGCβ1, or α-smooth muscle actin (1∶100 dilution; Abcam), followed by reaction with AlexaFluor goat anti-rabbit or anti-mouse IgG (1∶1000, Invitrogen), Dylight donkey anti-rabbit IgG (1∶100, Stratech Scientific), or biotinylated secondary antibodies.

Retinal flatmounts were prepared from paraformaldehyde-fixed eyes and stained with anti-βIII-tubulin (1∶150 dilution; Millipore) or anti-SMI32 (1∶500 dilution; Covance) antibodies. Stained RGCs were detected with biotin-, rhodamine-labeled secondary antibodies using confocal microscopy (Leica-TCS-SP5). For RGC counting, retinal flat mounts were divided into quadrants: superior, temporal, nasal, and inferior. Using the optic nerve head (ONH) as the origin, 3 standard regions that were distributed at a 1-mm interval along the radius (0.09 mm^2^) were selected from each quadrant: two from the peripheral region (2 mm from the ONH) and one from the intermediate region (1 mm from the ONH). 12 rectangular regions of each eye were photographed at 40× magnification with a confocal microscope (Leica TSC SPS confocal microscope).

For Toluidine Blue staining, mouse eyes were fixed in 2.5% glutaraldehyde and 2% paraformaldehyde, post-fixed in 2% osmium tetroxide in 1.5% potassium ferrocyanide, dehydrated, mid-sagittally sectioned, embedded in Epon-Araldite, and stained.

### Spectral domain optical coherence tomography

Retinal nerve fiber layer (RNFL) thickness was measured using a SD-OCT system (Bioptigen, NC). Pupils of anesthetized mice were dilated with topical application of 1% tropicamide. SD-OCT was performed using 100 horizontal raster and consecutive B-scan lines composed of 1200 A-scans. Total retinal thickness and RNFL thickness were analyzed using InVivoVue Diver 2.0 software (Bioptigen). Data were post-processed using a custom-made algorithm allowing additional filtering to only include RNFL thickness values between 5.4 and 24 microns in thickness. The 5.4–24 micron range was selected based on the average RNFL thickness ± 3 times the standard deviation described previously [32] for RNFL thickness of control mice. To validate the automated segmentation analysis, RNFL thickness was measured in 24 points of a 5×5 grid using the caliper tool provided by the Bioptigen software (fig. S1B).

### Paraphenylenediamine staining

Optic nerve cross-sections were prepared and axons were counted as previously described [33]. The myelin sheath of all axons, and the axoplasma of damaged axons were stained with paraphenylenediamine (PPD) and examined for glaucomatous damage. Sections of nerve between the orbit and chiasm were dissected, processed, embedded in resin, sectioned, and stained with PPD. To count axons, the optic nerve was outlined at 100× magnification and its cross-sectional area was automatically calculated (METAMORPH, Version 4.6r9, Universal Imaging, Downingtown, PA). Axons were counted manually and marked on images using the technique described previously [33]. The program tracked the total area counted and the total axon count for all images. The total counted area was 10% of the total nerve area. The final count was calculated and expressed as number of axons per optic nerve.

### IOP measurement

IOP measurements were acquired in anesthetized mice using a rebound tonometer (Tonolab) as described previously [34]. Five TonoLab readouts were averaged to obtain a single IOP value per eye.

### Ultrasound bio-microscopy (UBM)

UBM images were acquired and analyzed with a Vevo 770 scanner (VisualSonics), as previously described [35].

### Assessment of aqueous humor turnover

AqH turnover was measured noninvasively, using a previously published fluorophotometric technique [36]. Benzalkonium chloride (10 µl of a 0.02% solution in saline) was applied to the eye of anesthetized mice to permeabilize the cornea to fluorescein. After 5 minutes, 10 µl of a 0.02% fluoresce in saline solution was applied to the eye for 5 minutes. Images were captured from a focal plane intermediate between the iris and cornea using a microscope (SZX16, Olympus) equipped with an Olympus DP72 camera, a GFP filter, and acquisition software (cellSens Standard 1.2, Olympus). Average pixel intensity in the green channel was determined in an area of interest free of corneal defects using ImageJ. AqH clearance was determined by the decay constant calculated from the relative fluorescent intensity measured at 10-minute intervals for 60 minutes after a single fluorescein treatment.

### Scanning laser ophthalmoscopy

We previously developed a scanning laser ophthalmoscope (SLO) for confocal imaging of the mouse retina [37]. The SLO was used to record, at video rate, the width of the retinal arteries, visualized by intravenous (I.V.) injection of fluorescein sodium prior to, during, and after injection of 0.8 mg/kg sodium nitroprusside. To compute the width of the retinal arterioles, short segments (approximately 10 pixels in length) of the retinal arterioles were isolated from the recorded and re-aligned movies at fixed distances (measurements at 150 µm and 250 µm were averaged to yield one value/arteriole) from the optic nerve head. The width of each blood vessel segment was determined in MatLab as the width at which the pixel intensity dropped to 50% of the maximum (full width at half maximum, FWHM). The diameter of retinal arterioles (each 50 frame averages) was measured at baseline and after I.V. injection of 0.8 mg/kg sodium nitroprusside (in 50 µl of saline), a dose that was selected because it was associated with a similar drop in systemic blood pressure in WT and sGCα_1_^−/−^ mice. The relative change in diameter was calculated for each blood vessel. Injecting 50 µl of vehicle did not alter blood pressure or retinal arteriole diameter.

### In vivo hemodynamics

Mice were anaesthetized by IP injection with ketamine (100 mg/kg), fentanyl (50 µg/kg), and pancuronium (2 mg/kg); intubated; and mechanically ventilated (FiO_2_ = 1, 10 µl/g, 120 breaths per minute). A saline-filled catheter was inserted into the left carotid artery for infusion of saline (2 ml/h) and for measurement of mean arterial blood pressure (MAP) before, during and after I.V. injection of 0.8 mg/kg sodium nitroprusside. The sodium nitroprusside-induced decrease in MAP is expressed as % decrease from baseline.

### Statistical analysis for animal data

Statistical analyses (other than for the targeted gene association study, see below) were performed using Microsoft Excel or Stata 8.0. Normality of data was confirmed using the Shapiro-Wilk test. IOP, SLO, and MAP comparisons were performed using either student's t test when comparing 2 groups, 1-way ANOVA with Bonferroni post-hoc pairwise testing when groups were stratified by age, or multivariate linear regression to assess the ability of age to predict IOP. Least-squares regression analysis for exponential decay (Y = Ae^−kt^) was performed when assessing the change in aqueous fluorescein concentration (Y) over time (t), and exponential decay constants were compared using linear regression. Aqueous fluorescein concentrations and SD-OCT thickness were analyzed using 2-way repeated measures ANOVA, with post-hoc comparisons using 1-way ANOVA in circumstances of significant interaction *p*-values (e.g. for aqueous humor outflow rate measurements in 57-week-old mice). Data are presented as mean ± SD in the text and mean ± SEM in the figures. P<0.05 was considered significant.

### Association between GUCY1A3/GUCY1B3 single nucleotide polymorphisms (SNPs) and POAG in the Glaucoma Gene and Environment (GLAUGEN) study

The GWAS methods have been described previously [38]. Briefly, the GLAUGEN (**Glau**coma **g**enes and **en**vironment, dbGaP Study Accession **#**: phs000308.v1.p1) cohort included 976 POAG cases, defined as individuals with reproducible visual field defects correlating with clinical evidence of ON degeneration, and 1140 controls drawn from two longitudinal studies (the Nurses Health Study and the Health Professionals Follow-up study) and one clinic-based cohort from the Massachusetts Eye and Ear Infirmary. All participants were residents of the continental United States and were of European ancestry, confirmed by both self-identification and genetic markers. Genotyping was performed using the Illumina 660W-Quad-v1 platform at the Broad Institute and appropriate quality filters were applied to ensure a clean dataset. 495,132 SNPs passed quality control filters. POAG-SNP association in two soluble guanylate cyclase genes (GUCY1A3 and GUCY1B3) was analyzed. As these genes are adjacent, a list of SNPs within, between, and in a 50 kb window on either side of the genes was obtained using the UCSC Genome Browser [39]. These SNPs were entered into the SNAP proxy search to generate a list of all genotyped and tagging SNPs on the Illumina 660W-Quad platform, giving a total of 51 SNPs. Logistic regression for each gene to assess the association between individual SNPs and POAG was preformed using PLINK v1.07. The regression model included age, gender, race, study site, DNA source, DNA extraction method, and three eigenvectors. A sub-analysis was restricted to patients with paracentral visual field loss only (defined as having one or more paracentral scotomas and no peripheral visual field loss) leaving 175 cases and 1140 controls, and subsequently stratified according to gender (leaving 106 female cases and 682 female controls). SAS software was used to sort the results. To control for multiple comparisons (3 subgroups were analyzed: 1: POAG vs. control; 2: type of visual field loss; 3: gender), a Bonferroni correction of 3.3×10^−4^ (51 GUCY SNPs tested in 3 subsets of patients: 0.05/153 = 3.3×10^−4^) was applied to define statistical significance.

## Results

### Expression of sGC in the mouse and human eye

**Figure 1:**
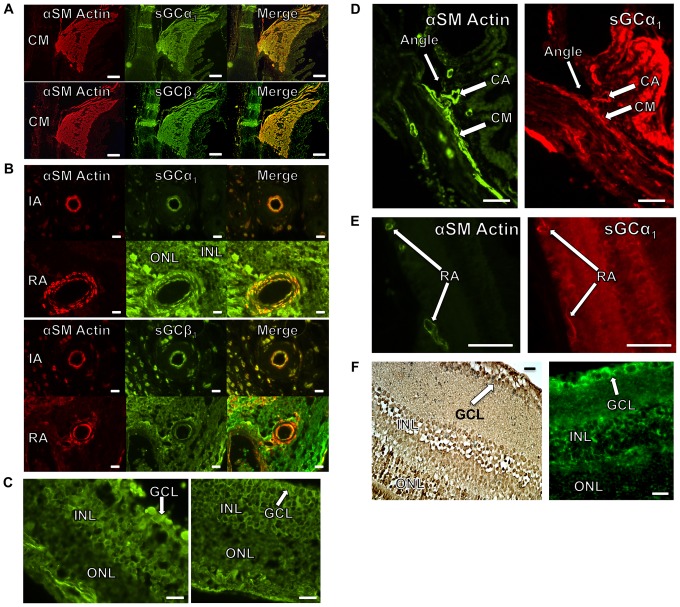
Localization of sGC α_1_ and β_1_ subunits in the human and murine eye. Panels **A–C** depict tissue sections from human eyes. Panels **D–F** depict tissue sections from mouse eyes. **A**: Ciliary muscle (CM), stained for α-smooth muscle actin (red), sGCα_1_ (green, **upper panel**), or sGCβ_1_ (green, **lower panel**). Both sGCα_1_ and sGCβ_1_ co-localized with α-smooth muscle actin in CM (yellow in merged images). Scale bars: 100 μm. **B**: An arteriole in the iris (IA) and an arteriole in the retina (RA) were stained for α-smooth muscle actin (red), sGCα_1_ (green, **upper panels**), or sGCβ_1_ (green, **lower panels**). Both sGCα_1_ and sGCβ_1_ co-localized with α-smooth muscle actin in the smooth muscle cell layer of arterioles in the iris and retina (yellow in merged images). ONL: outer nuclear layer, INL: inner nuclear layer. Scale bars: 20 μm. **C**: sGCα_1_ (**left panel**) and sGCβ_1_ (**right panel**) expression was detected histologically in the outer nuclear layer (ONL), inner nuclear layer (INL), and ganglion cell layer (GCL, white arrow) of the retina. sGCα_1_ and sGCβ_1_ are visualized by green fluorescence. Scale bars: 20 μm. **D**: Adjacent sections of a wild-type (WT) murine eye were stained for α-smooth muscle actin (green, **left panel**) or sGCα_1_ (red, **right panel**). sGCα_1_ co-localized with α-smooth muscle actin in ciliary muscle (CM) and in arterioles in the ciliary body (CA). The iridocorneal angle is indicated. Scale bars: 50 μm. **E**: Adjacent sections of a WT murine eye were stained for α-smooth muscle actin (green, **left panel**) or sGCα_1_ (red, **right panel**). sGCα_1_ co-localized with α-smooth muscle actin in retinal arterioles (RA). Scale bars: 50 μm. **F**: sGCα_1_ (**left panel**) and sGCβ_1_ (**right panel**) expression was detected histologically in the outer nuclear layer (ONL), inner nuclear layer (INL), and ganglion cell layer (GCL, white arrow) of the mouse retina. sGCα_1_ is visualized by brown peroxidase stain and sGCβ_1_ is visualized by green fluorescence. Scale bars: 20 μm.

Although expression of sGC has been detected in ocular tissue, including cultured human trabecular meshwork (TM) cells [20], human ciliary body and TM [40], drosophila photoreceptors [41], as well as rabbit [42], rat [43], and turtle [44] retina, detailed knowledge of spatial expression of the sGCα_1_β_1_ isoform in the eye is still lacking. Therefore, ocular sGCα_1_ and sGCβ_1_ localization was determined histologically in tissue sections of enucleated human and mouse eyes. sGCα_1_ and sGCβ_1_ are expressed in three anatomical sites that may be important for glaucoma. sGCα_1_ and sGCβ_1_ are abundantly expressed in the ciliary muscle (CM, fig. 1A and 1D), suggesting that sGC might modulate CM contractility and AqH outflow. sGC is also present in the smooth muscle cell layer of retinal blood vessels in the human and mouse eye (fig. 1B and 1E), implying that sGC regulates blood flow in the retina, just as it does in the systemic vasculature. Finally, sGCα_1_ and sGCβ_1_ are expressed in RGCs (fig. 1C and 1F), suggesting that sGC may directly regulate RGC function and/or viability.

### Retinal and optic nerve damage in sGCα_1_^−/−^ mice

**Figure 2:**
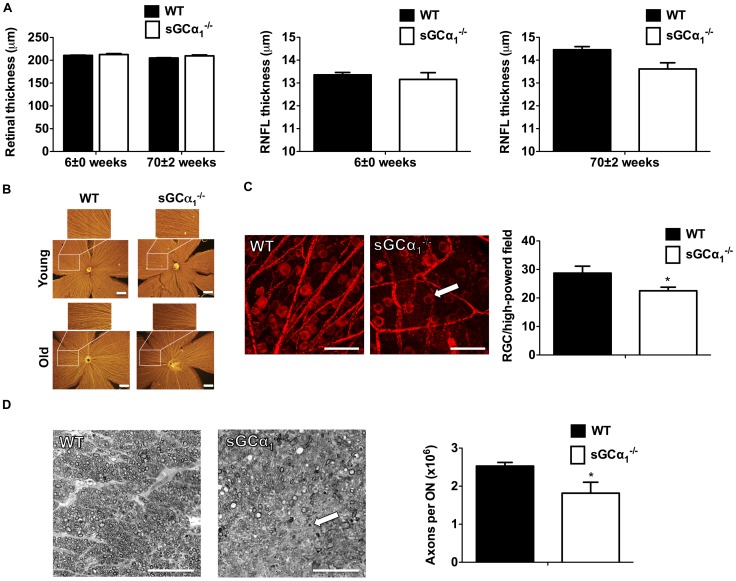
Retinal nerve fiber layer (RNFL) thinning and glaucomatous optic neuropathy in sGCα_1_^−/−^ mice. A : Quantitative analysis, assessed by SD-OCT (see also fig. S1), of total retinal thickness (**left panel**) and RNFL thickness, in young (6-week-old, **middle panel**) and old (70-week-old, **right panel**) wild-type (WT, *n* = 19 and 13, respectively) and soluble guanylate cyclase α_1_-deficient (sGCα_1_^−/−^) mice (*n* = 15 and 14, respectively; *P = 1.2×10^−2^). **B**: Representative whole-mount retinas from age-matched young (20-week-old) and old (56-week-old) WT and sGCα_1_^−/−^ mice, reacted with antibodies directed against SMI32, staining retinal nerve fibers yellow. Scale bars: 500 μm. **C**: Representative confocal images, taken at a similar distance from the optic nerve, of flat-mounted retinas isolated from age-matched 52-week-old WT and sGCα_1_^−/−^ mice that were reacted with antibodies directed against βIII Tubulin, and quantitative analysis of the number of RGCs/high-powered field (*n* = 8 and 7, respectively; *P = 3.6×10^−2^). A retinal ganglion cell (red) is indicated by an arrow. Scale bars: 20 μm. **D**: Representative cross sections through the optic nerve of 52-week-old WT and sGCα_1_^−/−^ mice stained with paraphenylenediamine, and quantitative analysis of the calculated number of axons/optic nerve (ON). The arrow indicates an injured area in the optic nerve, characterized by the absence of well-formed myelinated axons (*n* = 7 and 6, respectively; *P = 4.9×10^−2^). Scale bars: 25 μm.

To avoid the potentially confounding effects of systemic hypertension (a risk factor for POAG [1]) observed in male but not female sGCα_1_^−/−^ mice [31], [45], we included only female mice in our study that do not develop hypertension, even as they age [31]. Thickness of the retina and of the RNFL was measured using spectral-domain optical coherence tomography (SD-OCT), a technique previously shown to provide reproducible non-invasive measurements of RNFL thickness in mice [46]. Total retinal thickness was similar in sGCα_1_^−/−^ and age-matched wild-type (WT) mice, regardless of their age (fig. 2A and fig. S1). However, the RNFL was thinner in old sGCα_1_^−/−^ mice than in age-matched WT mice (fig. 2A and fig. S1). RNFL thickness did not differ in young, age-matched sGCα_1_^−/−^ and WT mice (fig. 2A and fig. S1), suggesting that abnormal embryonic development does not contribute to the observed RNFL thinning associated with sGCα_1_-deficiency.

To further characterize RNFL thinning in sGCα_1_^−/−^ mice, retinal whole mounts from WT and sGCα_1_^−/−^ mice were stained with either an anti-SMI32 antibody that stains nerve fibers or an anti-βIII tubulin antibody that stains both ganglion cells and nerve fibers. SMI32 staining revealed a loss of nerve fibers in whole-mount retinas isolated from old but not young sGCα_1_^−/−^ mice (fig. 2B). Moreover, a lower number of RGCs were detected in old sGCα_1_^−/−^ mice than in age-matched WT mice, a finding that was confirmed by immunohistochemistry in retinal flat-mounts (fig. 2C).

Because POAG is typically characterized by optic nerve damage, we investigated the impact of sGCα_1_-deficiency on total axon counts in the optic nerve, visualized using paraphenylenediamine staining. The number of axons in twelve-month-old sGCα_1_^−/−^ mice was less than in age-matched WT mice (fig. 2D). Together, these results indicate that, in mice, impaired NO-cGMP signaling results in optic neuropathy, an important feature of POAG.

### Age-related intraocular pressure elevation in sGCα_1_^−/−^ mice

Because IOP is considered the most important risk factor for POAG, IOP was measured non-invasively in young and old WT and sGCα_1_^−/−^ mice. No difference in IOP was detected between young sGCα_1_^−/−^ and age-matched WT mice. In contrast, IOP was higher in old sGCα_1_^−/−^ mice than in age-matched WT mice (fig. S2A).

**Figure 3:**
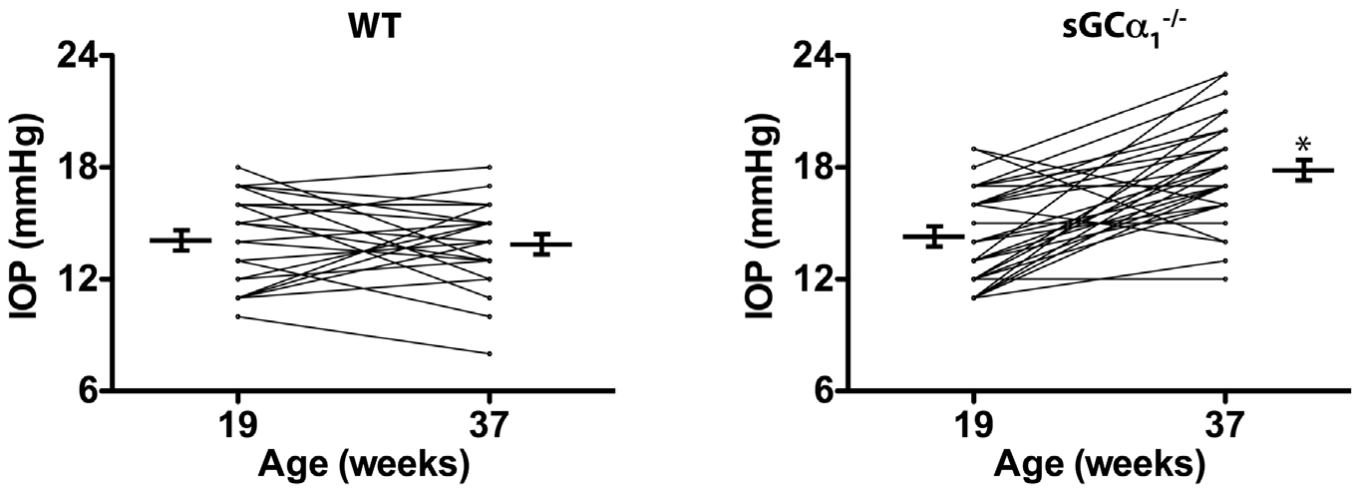
Intraocular pressure (IOP) increases with age in sGCα_1_^−/−^ mice. IOP, measured serially at 2 time points (19±1 and 37±3 weeks) in eyes from age-matched wild-type (WT, **left panel**) and soluble guanylate cyclase α_1_-deficient (sGCα_1_^−/−^) mice (**right panel**). While IOP remained stable in WT mice as they aged from 19 to 37 weeks (14±2 to 14±2 mmHg; *n* = 25; *P* = 0.67), IOP increased in sGCα_1_^−/−^ mice (14±2 to 18±3 mmHg; *n* = 37; *P = 1.9×10^−8^).

To further investigate the age-dependency of increased IOP associated with sGCα_1_-deficiency, we performed longitudinal measurements of IOP in age-matched sGCα_1_^−/−^ and WT mice. IOP increased in sGCα_1_^−/−^ but not in WT mice as they aged from 19 to 37 weeks (fig. 3). Similar results were obtained in separate validation cohorts of WT and sGCα_1_^−/−^ mice (fig. S2B and C). Together, these results demonstrate that sGCα_1_-deficiency is associated with a subtle age-dependent IOP increase in mice.

### sGCα_1_ deficiency does not alter anterior segment morphology

**Figure 4:**
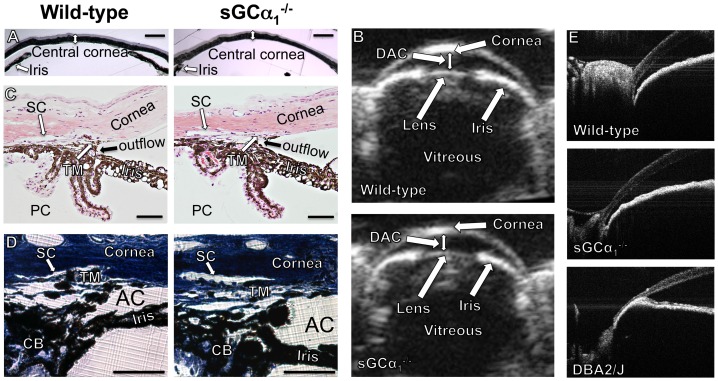
Morphology of the cornea and anterior segment does not differ in WT and sGCα_1_^−/−^ mice. A : Representative light microscopic images of the central cornea in wild-type (WT, left) and soluble guanylate cyclase α_1_-deficient mice (sGCα_1_^−/−^, right). Central corneal thickness (CCT) was similar in age-matched WT and sGCα_1_^−/−^ mice. Double arrows: CCT. Scale bar: 200 μm. **B**: Representative ultrasound biomicroscopy images of the ocular anterior segment, obtained in WT (**upper panel**) and sGCα_1_^−/−^ mice (**lower panel**). The cornea, iris, vitreous, and lens are evident. Depth of the anterior chamber (DAC, double arrow) did not differ in WT and sGCα_1_^−/−^ mice. **C** and **D**: Representative light microscopic images of paraffin sections stained with hematoxylin and eosin (C, scale bar: 100 μm) or Toluidine Blue (D, scale bar: 50 μm) containing the iridocorneal angles of 12-month-old WT (**left panels**) and sGCα_1_^−/−^ mice (**right panels**). Location of the cornea, iris root, ciliary body (CB), anterior chamber (AC), and posterior chamber (PC) are indicated. **E**: Representative SD-OCT images of the ocular anterior segment, obtained in a 12-month-old wild-type mouse (**upper panel**), a 12-month-old sGCα_1_^−/−^ mouse (**middle panel**), and a 12-month-old DBA2/J mouse in which the angle is closed (**lower panel**). SD-OCT revealed no morphological abnormalities in sGCα1^−/−^ mice that would suggest a closed angle as detected in old DBA2/J mice. See also **Movies S1, S2, S3.**

Central corneal thickness (CCT) is a risk factor for the conversion from ocular hypertension to POAG, making CCT measurements an integral component in the management of patients diagnosed with glaucoma [5]. In addition, CCT influences the measurement of IOP via rebound tonometry [47]. CCT, measured histologically, did not differ in age-matched 38±3-week-old WT and sGCα_1_^−/−^ mice (87±7 and 87±9 μm, respectively; *n* = 8 each; *P* = 0.90) with normal and elevated IOP, respectively (14±1 versus 21±1 mmHg; *P* = 5.9×10^−7^). Therefore, sGCα_1_-deficiency does not affect CCT and abnormal CCT is not responsible for the elevated IOPs seen in old sGCα_1_^−/−^ mice (fig. 4A).

To exclude the possibility that anatomical abnormalities impairing AqH drainage, such as those observed in DBA/2J mice [48] or in mice deficient in cytochrome P4501B1 (Cyp1b1^−/−^ mice) [49], underlie the elevated IOP observed in older sGCα_1_^−/−^ mice, we examined the morphology of the iridocorneal angle in 12-month-old WT and sGCα_1_^−/−^ mice with normal and elevated IOP, respectively (15.8±1.2 and 19.1±1.7 mmHg; *n* = 3 each; *P* = 0.041). Morphologically, eyes of sGCα_1_^−/−^ mice appeared normal, with a clear anterior chamber revealing complex iris detail and small round pupils. Depth of the anterior chamber (DAC) was assessed using *in vivo* ultrasound biomicroscopy. DAC did not differ in 56±3-week-old WT and sGCα_1_^−/−^ mice (0.34±0.01 and 0.33±0.01 mm in *n* = 4 and 6, respectively; *P* = 0.26) with IOPs of 16±2 and 19±2 mmHg, respectively (*P* = 4.3×10^−2^, fig. 4B). In addition, ultrasound biomicroscopy revealed no morphological abnormalities in sGCα_1_^−/−^ mice that could account for the elevated IOP measurements. Histological analyses revealed no apparent iridocorneal abnormalities in 12-month-old sGCα_1_^−/−^ mice, including a normal ciliary body, a well-defined trabecular meshwork, and a patent Schlemm's canal (fig. 4C and D). Importantly, SD-OCT analysis of the iridocorneal angle, capable of distinguishing between an open and a closed angle (as in old DBA2/J mice), did not reveal any evidence for angle-closure in 12-month-old sGCα_1_^−/−^ mice (fig. 4E and movies S1, S2, S3). Taken together, these data suggest that sGCα_1_-deficiency does not alter the morphology of the anterior segment of the eye and that angle closure does not underlie the elevated IOP observed in old sGCα_1_^−/−^ mice.

### Decreased aqueous humor turnover in sGCα_1_^−/−^ mice

**Figure 5:**
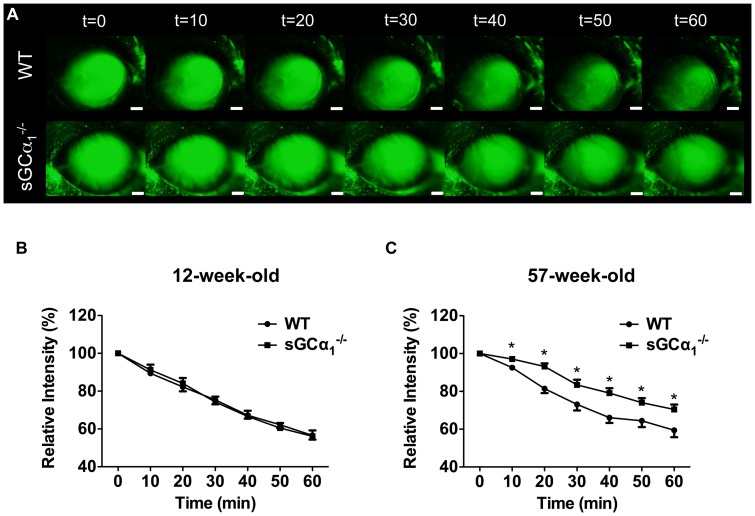
Decreased aqueous humor outflow rate in sGCα_1_^−/−^ mice. A : Shown is a representative series of images captured from 57-week-old wild-type (WT) and soluble guanylate cyclase α_1_-deficient (sGCα_1_^−/−^) mice at 10-minute intervals after application of fluorescein. Scale bars: 2 mm. **B**: In 12-week-old WT and sGCα1^−/−^ mice with similar IOPs (16±2 and 16±1 mmHg in *n* = 9 and 8, respectively; *P* = 0.91) the rate of AqH outflow did not differ, as shown by similar relative fluorescent intensities at all time points measured (*P* = 0.99) and similar exponential decay constants (0.0098 min^−1^ (*r* ^2^ = 0.997) and 0.0096 min^−1^ (*r* ^2^ = 0.998), respectively; *P* = 0.12). **C**: IOP was greater in 57-week-old sGCα1^−/−^ mice than in age-matched WT mice (18±2 mmHg and 16±1, respectively; *n* = 10 each; *P* = 0.043), and AqH clearance was delayed in sGCα1^−/−^ mice, as shown by a lower exponential decay constant (0.0058 min^−1^ (*r* ^2^ = 0.972) versus 0.0092 min^−1^ (*r* ^2^ = 0.976), respectively; *P* = 0.0056) and higher fluorescent intensities at all time points measured. *P = 0.033, 0.0006, 0.024, 0.0029, 0.035, 0.025, between WT and sGCα_1_^−/−^ mice at 10, 20, 30, 40, 50, and 60 minutes, respectively.

IOP is determined by the balance between AqH production by the ciliary body and the rate of AqH outflow through the filtration angle. The AqH outflow rate was assessed using a fluorophotometric technique in age-matched WT and sGCα_1_^−/−^ mice (fig. 5A). Monitoring decay of fluorescence provides a noninvasive index of AqH dynamics in the mouse eye [36]. Twelve-week-old WT and sGCα_1_^−/−^ mice had similar IOPs and no difference in the rate of AqH outflow (fig. 5B). In contrast, AqH clearance was less in 57-week-old sGCα_1_^−/−^ mice with elevated IOP than in age-matched WT mice (fig. 5C). These data indicate that there is an age-related decrease in AqH outflow in sGCα_1_^−/−^ mice.

### Retinal arterial dysfunction in sGCα_1_^−/−^ mice

**Figure 6:**
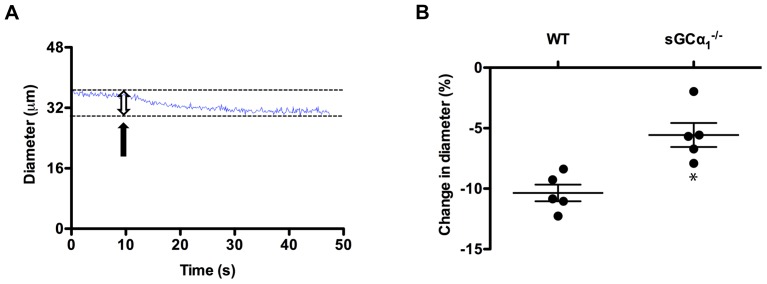
Retinal vascular dysfunction in sGCα_1_^−/−^ mice. A : Representative trace depicting the diameter in one segment of a retinal arteriole in a wild-type (WT) mouse before, during (arrow), and after injection of the NO-donor compound sodium nitroprusside. Dashed lines indicate the diameter before and after sodium nitroprusside injection. **B**: Quantitative analysis of the change in diameter (double arrow in fig. 6A) induced by injection of 0.8 mg/kg sodium nitroprusside in WT and sGCα_1_^−/−^ mice. *n* = 5 mice (3–4 arterioles per mouse were assessed, see also fig. S3). *P = 4.1×10^−3^.

We previously reported that systemic vascular dysfunction, a potential etiologic factor for POAG [7], [8], [9], is manifest in the aorta [16], femoral arteries [16], carotid arteries [50], and mesenteric arteries [45] of both male and female sGCα_1_^−/−^ mice. To assess retinal vascular function [9], we measured retinal arterial diameter using in vivo laser ophthalmoscopy (SLO, fig. S3A and B). Treatment with an NO-donor compound decreased blood pressure similarly in female WT and sGCα_1_^−/−^ mice (59±2% and 60±3%, respectively, *n* = 5 each; *P* = 0.37). However, retinal arterial diameter decreased more in female WT than in sGCα_1_^−/−^ mice (fig. 6 and fig. S3C). An altered retinal hemodynamic response to a decrease in blood pressure indicates that vascular reactivity in the retinal vasculature is abnormal in sGCα_1_^−/−^ mice.

### Association between a genetic locus containing the genes encoding the α_1_ and β_1_ subunits of sGC and POAG

To explore the contribution of sGC to human POAG pathogenesis, we evaluated 51 single nucleotide SNP located throughout the genomic region that includes *GUCY1A3* and *GUCY1B3* for their association with POAG in humans. *GUCY1A3* and *GUCY1B3* encode the sGCα_1_ (inactivated in sGCα_1_^−/−^ mice) and sGCβ_1_ subunits, respectively, that constitute the predominant sGC isoform in most types of tissues [51]. We performed a gene association study among POAG cases and controls from the Glaucoma Genes and Environment (GLAUGEN) study [38]. None of the tested SNPs were associated with POAG overall (table 1).

**Table 1:** Association between *GUCY1A3/GUCY1B3* single nucleotide polymorphisms (SNPs) and POAG in the Glaucoma Gene and Environment (GLAUGEN) study.

Gender	VF loss	MA	Top SNP	P value	OR	95% CI	Chr	N cases	N controls
all	any	A	rs9992550	0.04271	1.43	(1.01, 2.02)	4	976	1140
women	any	A	rs11722059	0.02734	1.29	(1.03,1.61)	4	570	682
men	any	G	rs13115024	0.05038	0.61	(0.38, 1.00)	4	406	458
all	paracentral	A	rs11722059	0.00406	1.50	(1.14, 1.98)	4	175	1140
***women***	***paracentral***	***A***	***rs11722059***	***0.00031***	***1.90***	***(1.34, 2.69)***	***4***	***106***	***682***
men	paracentral	A	rs4691842	0.01468	1.62	(1.10, 2.40)	4	69	458

Most significant single nucleotide polymorphisms (SNPs) stratified by gender and type of visual field (VF) loss. 51 SNPs were analyzed within the GUCY1A3/GUCY1B3 locus and 50 kb upstream and downstream of the region. A Bonferroni correction of 3.3×10^−4^, correcting for analyzing 51 SNPs and 3 subgroups, was applied to define statistical significance. The top SNP (rs11722059) reached significance in women with paracentral visual field (VF) loss (P value of 3.1×10^−4^, bold and italicized). MA: minor allele; OR: multivariable odds ratio associated with each minor allele dose, generated by multiple logistic regression analyses adjusting for age, gender, study site, DNA source, DNA extraction method, and three eigenvectors; CI: confidence interval; Chr: chromosome; N: number.

We next hypothesized that POAG in sGCα_1_^−/−^ mice (with systemic and retinal vascular dysfunction) shares a common etiology with POAG in patients with paracentral visual field loss, a subtype of POAG previously suggested to be associated with vascular dysregulation [10]. Therefore, we focused our analysis on a subpopulation of POAG patients with initial paracentral scotomas and no peripheral visual field loss. Given the previously described interactions between NOS3 and female gender [26] in POAG, we analyzed men and women separately. The SNP rs11722059, located in the *GUCY1A3/GUCY1B3* intergenic region, was significantly associated with POAG characterized by paracentral visual field loss in women (*P* = 3.1×10^−4^, *OR* = 1.90, *CI* = 1.34–2.69; table 1). Replication of the candidate gene association study is required to confirm the result from this candidate gene association study. Nevertheless, tentative identification of a risk variant located in the locus encoding sGCα_1_ and sGCβ_1_, both expressed in ocular tissue (fig. 1), highlights the potential relevance of sGC in the pathogenesis of POAG.

## Discussion

In this study, we report that sGCα_1_^−/−^ mice develop POAG, characterized by RNFL thinning, loss of RGC's, and optic neuropathy in the setting of an open iridocorneal angle. An age-dependent decrease in AqH turnover suggests that an increase in outflow resistance underlies the elevated IOP observed in old sGCα_1_^−/−^ mice. In addition, our findings suggest that impaired NO-cGMP signaling results in retinal vascular dysfunction, a possible contributor to the pathophysiology of POAG. Importantly, the relevance of sGCα_1_^−/−^ mice as a new animal model for POAG is highlighted by the tentative identification of a genetic association between the genetic locus encoding the α_1_ and β_1_ subunits of sGC and a POAG subtype thought to be associated with vascular dysregulation.

The RNFL contains the RGC axons that converge to form the optic nerve. Glaucoma is characterized by loss of axons and death of RGCs, which can be detected as thinning of the RNFL. Retinal damage, characterized by thinning of the RNFL and loss of RGCs in old sGCα_1_^−/−^ but not age-matched WT mice was demonstrated via SD-OCT and histologic examination. In addition, the number of optic nerve axons was significantly lower in twelve-month-old sGCα_1_^−/−^ mice than in age-matched WT mice, indicating optic neuropathy associated with sGCα_1_-deficiency. The observation that RNFL thickness did not differ in young WT and sGCα_1_^−/−^ mice, neither of which had high IOPs, suggests that lack of sGCα_1_ does not directly affect ganglion cell viability during embryonic development or during the first weeks after birth.

Several risk factors for POAG have been suggested [2]. Elevated IOP is the best characterized risk factor but may not explain all POAG risk. It is becoming increasingly clear that compounds that do not lower IOP dramatically but that have properties that address the underlying glaucomatous disease process may be suitable therapeutic agents [52], [53]. For example, brimonidine, an α_2_-adrenergic receptor agonist postulated to modulate vascular reactivity in an NO-dependent manner [54], corrected the retinal vasculopathy in patients with normotensive glaucoma and retinal vascular dysregulation [55]. A randomized clinical trial revealed that brimonidine was superior to timolol in stabilizing visual field deterioration despite producing only a modest IOP-lowering effect (∼1 mmHg) similar to that of timolol [56]. Together, these data suggest that strategies, other than lowering IOP, such as those targeting vascular dysfunction, may be efficacious treatments for glaucoma [57]. It is important to emphasize that, even though in the majority of glaucoma clinical drug trials reduction of IOP is the primary efficacy endpoint, the United States Food and Drug Administration has expressed interest in drugs that prevent progression of glaucomatous damage without necessarily lowering IOP [52], [53]. Nonetheless, benefit-to-risk ratio will likely continue to be determined by drug efficacy and health risk assessment from clinical trials in comparison to the benchmark, IOP lowering drugs [52].

Our data demonstrates that impaired NO-cGMP signaling results in elevated IOP. The observation that IOPs are similar in young WT and sGCα_1_^−/−^ mice but increase with age in sGCα_1_^−/−^ but not WT mice indicates that elevated IOP seen in sGCα_1_^−/−^ mice depends on age, an important risk factor for POAG [1]. Similarly modest changes in IOP impact POAG risk in humans, highlighting the potential biological significance of the increase in IOP in sGCα_1_^−/−^ mice [58]. For example, a 2 mmHg difference in IOP distinguished between progression and non-progression in glaucoma patients [58], [59], [60]. Additional longitudinal studies are required to further characterize the exact timeframe of the increase in IOP associated with sGCα_1_-deficiency. Examination (by histology, ultrasound, and SD-OCT) of the iridocorneal angle and anterior chamber of sGCα_1_^−/−^ mice with elevated IOP did not reveal any evidence of morphological abnormalities of the peripheral cornea or outflow pathway illustrating that the outflow pathway was not physically obstructed (a characteristic of closed-angle glaucoma). The rate of AqH outflow dynamics, assessed via a previously reported non-invasive method [36] that will require validation using a more reliable method [61], was similar in young WT and sGCα_1_^−/−^ mice but impaired in old sGCα_1_^−/−^ mice with elevated IOPs suggesting that an increase in outflow resistance (indicative of POAG) may contribute to the IOP increase in sGCα_1_^−/−^ mice. The mechanism underlying the age-dependency of the effect of sGC-deficiency on AqH outflow dynamics remains to be determined.

The abundant expression of sGCα_1_ and sGCβ_1_ in the CM, together with the established role of sGC in mediating smooth muscle cell relaxation [16], [17], suggests that sGC can alter outflow by regulating smooth muscle-like contractility of the longitudinal fibers of the CM. The contractile state of smooth muscle tissue is the target of Rho-kinase inhibitors, currently being tested in clinical trials for glaucoma and ocular hypertension [62]. It is conceivable that AqH drainage is similarly influenced by impaired NO-cGMP signaling and enhanced RhoA-signaling: both could result in augmented contractility of the longitudinal fibers of the CM, thereby potentially decreasing AqH drainage and increasing IOP [63]. However, the possibility remains that sGCα_1_-deficiency affects AqH production or that changes in Schlemm's canal cell or TM cell volume, previously reported to be modulated by NO-cGMP signaling [64], contribute to the altered AqH dynamics in sGCα_1_^−/−^ mice.

The hypothesis that retinal vascular dysfunction contributes to the glaucomatous phenotype in sGCα_1_^−/−^ mice was based on the observed peripheral vascular dysfunction in sGCα_1_^−/−^ mice and on the association of a genetic variant in the locus containing the sGCα_1_ and sGCβ_1_ genes with glaucomatous paracentral vision loss in women, a subtype of POAG previously postulated to be associated with ocular vascular dysregulation [10]. To test whether retinal vascular function is affected by sGCα_1_-deficiency, the retinal vasculature was visualized via SLO and the diameter of retinal arteries was measured at baseline and after treatment with the NO donor sodium nitroprusside. We previously demonstrated that the ability of a low dose of sodium nitroprusside to reduce blood pressure was attenuated in sGCα_1_^−/−^ mice, reflecting the systemic vascular dysfunction associated with impaired NO-cGMP signaling [31]. In contrast, a high dose of sodium nitroprusside reduced blood pressure similarly in sGCα_1_^−/−^ and WT mice [31], a mechanism that involves activation of sGCα_2_β_1_ [17]. Systemic administration of sodium nitroprusside at a dose that decreased blood pressure similarly in WT and sGCα_1_^−/−^ mice, thereby avoiding the potential confounding impact on retinal vascular diameter of differences in systemic blood pressure between WT and sGCα_1_^−/−^ mice, induced vasoconstriction of the retinal vasculature in both WT and sGCα_1_^−/−^ mice. Vasoconstriction likely results from baroreflex activation in response to an acute drop in blood pressure or may be mediated by the renin-angiotensin system, as previously described in other vascular beds in response to circulatory shock [65]. Importantly, the decrease in retinal arterial diameter upon NO administration was less apparent in sGCα_1_^−/−^ than in WT mice. The observation that the retinal hemodynamic response to a decrease in blood pressure is abnormal in sGCα_1_^−/−^ mice, suggests that impaired NO-cGMP signaling is associated with retinal vascular dysfunction, just as it is with systemic vascular dysfunction. In summary, sGCα_1_-deficiency may result in optic neuropathy via a variety of mechanisms, either by increasing IOP and/or by impairing retinal blood flow. In addition, we cannot exclude the possibility that impaired NO-sGC signaling sensitizes RGCs to small increases in IOP that may not injure WT RGCs [66]. Also, we recognize that additional work is necessary to examine the impact of gender on the development of POAG in sGCα_1_^−/−^ mice, especially in light of observations suggesting that gender may play an important role in the pathogenesis of POAG [26].

Although several mechanically- and chemically-induced models of ganglion cell death and glaucoma have been developed in mice [67], [68] to date, only a few spontaneously-occurring murine models of glaucoma that do not involve ocular manipulation have been described. As they age, DBA/2J mice develop a progressive form of secondary angle closure glaucoma [48]. TG-MYOC^Y437H^ mice that overexpress a mutant form of the human myocilin (MYOC) gene mimics human juvenile open-angle glaucoma and is considered a model of POAG [69]. However, mutations in MYOC account for only a minority of POAG cases. Cyp1b1^−/−^ mice develop angle dysgenesis that mimics the congenital version of open-angle glaucoma but these mice do not develop elevated IOP [49]. More recently, transiently elevated IOP [70], optic nerve damage [71], and decreased AqH outflow [72] were described in mice with a targeted mutation in the type I collagen gene. Our data suggests that sGCα_1_^−/−^ mice are an additional model of POAG that may be useful to study mechanisms of IOP regulation and the role of vascular dysfunction in the etiology of POAG.

Linkage analysis and genome-wide association studies have identified several genes associated with POAG, a disease with significant heritability [73], [74]. Our identification, in an exploratory genetic analysis aimed at investigating the relevance of our mouse model in human POAG, of a variant (rs11722059) in the *GUCY1A3/GUCY1B3 locus* significantly associated with a subpopulation of POAG patients characterized by paracentral scotomas, is a step towards understanding the contribution of sGC to the etiology of this important subtype of POAG. Interestingly, rs11722059 is in linkage disequilibrium (D' = 0.86; r^2^ = 0.67) with another *GUCY1A3/GUCY1B3* SNP (rs13139571) that was recently associated with blood pressure in 200,000 individuals of European decent from the International Consortium for blood pressure [75]. Overall, these results identify a role for sGCα_1_β_1_ in the POAG phenotype characterized by vascular dysregulation and paracentral visual field loss, and suggest a possible mechanistic link between blood pressure and POAG. Together with the previously reported association between vascular dysfunction and POAG [8], [55], and with the finding that sGCα_1_-deficiency is associated with both systemic and retinal vascular dysfunction, the results of our candidate gene association study raises the possibility that retinal vascular dysfunction contributes to the development of POAG associated with impaired NO-cGMP signaling.

While the focus of this study was to categorize a new murine model of POAG and identify a well-characterized signaling mechanism (NO-cGMP signaling) as centrally involved in the etiology of POAG, rather than to formally report on a new genetic association, we recognize that the human genetic association described requires confirmation in a replication cohort. Because recruitment ascertainment typically targets patients more likely to have peripheral visual field defects, there are currently no other available POAG cohorts of sufficient size with paracentral scotoma information that could be used for replication. For example, only 26 of 2170 cases in the NEI Glaucoma Human Genetics Collaboration presented with initial paracentral loss [76]. In spite of this limitation, we believe the genetic association data provided highlights the significance of sGC-signaling in the etiology of POAG and will motivate investigators to identify cohorts to replicate the GLAUGEN finding.

In humans, several mechanisms have been identified that can impair NO-cGMP signaling, potentially contributing to the development of POAG. For example, a candidate gene association study in 527 incident cases and 1543 controls revealed interactions between NOS3 gene variants, potentially affecting expression and/or activity of NOS3 (and, ultimately, sGC enzyme activity), and high tension POAG in females [26]. Alternatively, the mechanism by which increased oxidative stress results in POAG [77] may involve direct oxidation and inactivation of sGC [78]. NO- and heme-independent sGC activators, capable of activating oxidized sGC, such as cinaciguat [79], are available and are being tested in the clinic for a variety of cardiovascular diseases [80]. Other potential therapeutic targets include proteins that control GMP levels and which are abundantly present in the eye (including cGMP-catabolizing phosphodiesterases [81] and particulate guanylate cyclases [82]). Pharmacological modulation of the activity of these proteins may increase outflow, attenuate retinal arterial dysfunction, and prevent disease progression.

## Conclusions

Our results demonstrate that impairing a well-characterized signaling pathway (NO-cGMP signaling) results in optic neuropathy, possibly by modulating IOP and retinal vascular function. Our data identify sGCα_1_^−/−^ mice as a novel translational animal model for POAG, providing a tool for investigators to study the pathogenesis of POAG and to test new strategies for disease prevention. Furthermore, we identified a genomic locus containing the sGCα_1_ and sGCβ_1_ genes that is associated with glaucoma with paracentral visual field loss in women. Together, these findings identify sGC as a potential therapeutic target to treat POAG and may inform the clinical development of existing cGMP-elevating therapeutic compounds for treating glaucomatous retinal injury.

## Supporting Information

Figure S1 **SD-OCT analysis of retinal nerve fiber layer thickness in sGCα_1_^−/−^ mice. A**: Representative heat maps of absolute total retinal thickness and RNFL thickness in 12 month-old wild-type (WT) and soluble guanylate cyclase α_1_-deficient (sGCα_1_^−/−^) mice. No differences in total retinal thickness were detected between sGCα_1_^−/−^ and WT mice (fig. 2A). RNFL thickness was thinner in sGCα_1_^−/−^ mice than in WT mice, as demonstrated by the darker blue colors in the sGCα_1_^−/−^ RNFL heat map (see also fig. 2A). Color scale varies between 1.53 (black) and 247.93 (white) μm. See fig. 2A for quantitative data. **B**: Validation of the automated segmentation analysis. The manually determined RNFL thickness data recapitulated the automated segmentation analysis and confirmed that the RNFL is thinner in old in sGCα_1_^−/−^ mice than in old WT mice. **Left panel**: Representative en-face image of the retina acquired using SD-OCT imaging (left), showing the 24 points (marked by X) of a 5×5 grid at which RNFL thickness was measured, and B-scans (right, A-E) showing placement of the 24 calipers used to measure RNFL thickness. **Middle panel**: mean RNFL thickness measured in all 24 pre-defined points in WT (upper number) and sGCα_1_^−/−^ mice (lower number). **Right panel**: mean RNFL thickness (as averages of RNFL thickness measured manually in 24 points), assessed using a 2-way repeated measures ANOVA, did not differ in 6-week-old sGCα_1_^−/−^ and age-matched WT mice (*n* = 15 and 20, respectively; *P* = 0.29), but was thinner in 70-week-old sGCα_1_^−/−^ mice than in age-matched WT mice (*n* = 17 each; *P = 1.4×10^−2^).
(TIF)

Figure S2 **Intraocular pressure (IOP) increases with age in sGCα_1_^−/−^ mice. A**: IOP in young (15±6-week-old) and old (39±14-week-old) wild-type (WT) and soluble guanylate cyclase α_1_-deficient (sGCα_1_^−/−^) mice. No statistically significant difference in IOP was detected between young sGCα_1_^−/−^ and age-matched WT mice (*n* = 61 and 112, respectively; *P* = 0.08). In contrast, IOP was higher in old sGCα_1_^−/−^ mice than in age-matched WT mice (*n* = 468 and 140, respectively). *P = 2.9×10^−7^ vs. 39±14-week-old WT. **B**: IOP in a second independent cohort of WT eyes (*n* = 27) measured serially at 2 time points. IOP was 15±2 and 15±1 mmHg in 30±0 and 62±0-week-old WT mice, respectively. *P* = 0.93. **C**: IOP in a second independent cohort of sGCα_1_^−/−^ eyes (*n* = 41) measured serially at 2 time points. IOP increased from 15±2 to 19±2 mmHg in 26±4 and 73±5-week-old sGCα_1_^−/−^ mice, respectively. *P = 4.8×10^−16^.
(TIF)

Figure S3 **Fluorescein angiography in WT and sGCα_1_^−/−^ mice. A**: Serial images (A–G) of fluorescein angiography between 1 and 3 seconds after I.V. injection of fluorescein. Retinal arterioles (arrows in **panel A**) were identified based on earlier filling with fluorescein than veins (arrows in **panel E**). **B**: Image of fluorescein angiography. The width of the arterioles was determined (boxes) at fixed distances from the optic nerve head (inner dashed ring  = 150 µm; outer dashed ring  = 250 µm). **C**: Quantitative analysis of the diameter of retinal arterioles in wild type (WT, **left panel**) and soluble guanylate cyclase α_1_-deficient mice (sGCα_1_^−/−^, **middle panel**) before and after a challenge with 0.8 mg/kg sodium nitroprusside. Baseline diameter of retinal arterioles was similar in WT and sGCα_1_^−/−^ mice (*P* = 0.40). Injection of 0.8 mg/kg sodium nitroprusside reduced the diameter of retinal arterioles in both WT (*P = 7.8×10^−12^) and sGCα_1_^−/−^ mice (*P = 1.7×10^−6^). The change in diameter induced by injection of sodium nitroprusside was more pronounced in WT than in sGCα_1_^−/−^ mice (**right panel**, *P = 9.0×10^−6^). *n* = 17 and 15 WT and sGCα_1_^−/−^ arterioles, respectively; 3–4 arterioles/mouse from 5 mice, each. See also fig. 6B for data analysis per mouse.
(TIF)

Movie S1 Representative movies of SD-OCT analyses of the iridocorneal angle in age matched 12-month old wild-type (WT, **movie S1**) and soluble guanylate cyclase α_1_-deficient mice (**movie S2**). To demonstrate the ability of SD-OCT to discriminate between open and closed angles we imaged a closed angle in a 12-month-old DBA/2J mouse with elevated IOP (**movie S3**). Movies are representative examples of images observed in at least five mice per group in two replicate experiments.
(MOV)

Movie S2 Representative movies of SD-OCT analyses of the iridocorneal angle in age matched 12-month old wild-type (WT, **movie S1**) and soluble guanylate cyclase α_1_-deficient mice (**movie S2**). To demonstrate the ability of SD-OCT to discriminate between open and closed angles we imaged a closed angle in a 12-month-old DBA/2J mouse with elevated IOP (**movie S3**). Movies are representative examples of images observed in at least five mice per group in two replicate experiments.
(MOV)

Movie S3 Representative movies of SD-OCT analyses of the iridocorneal angle in age matched 12-month old wild-type (WT, **movie S1**) and soluble guanylate cyclase α_1_-deficient mice (**movie S2**). To demonstrate the ability of SD-OCT to discriminate between open and closed angles we imaged a closed angle in a 12-month-old DBA/2J mouse with elevated IOP (**movie S3**). Movies are representative examples of images observed in at least five mice per group in two replicate experiments.
(MOV)
