# Supplementary figures and images for: Soluble Guanylate Cyclase α1–Deficient Mice: A Novel Murine Model for Primary Open Angle Glaucoma
Source: PLoS One. 2013 Mar 20;8(3):e60156. doi: 10.1371/journal.pone.0060156 (PMC3603933; doi:10.1371/journal.pone.0060156)

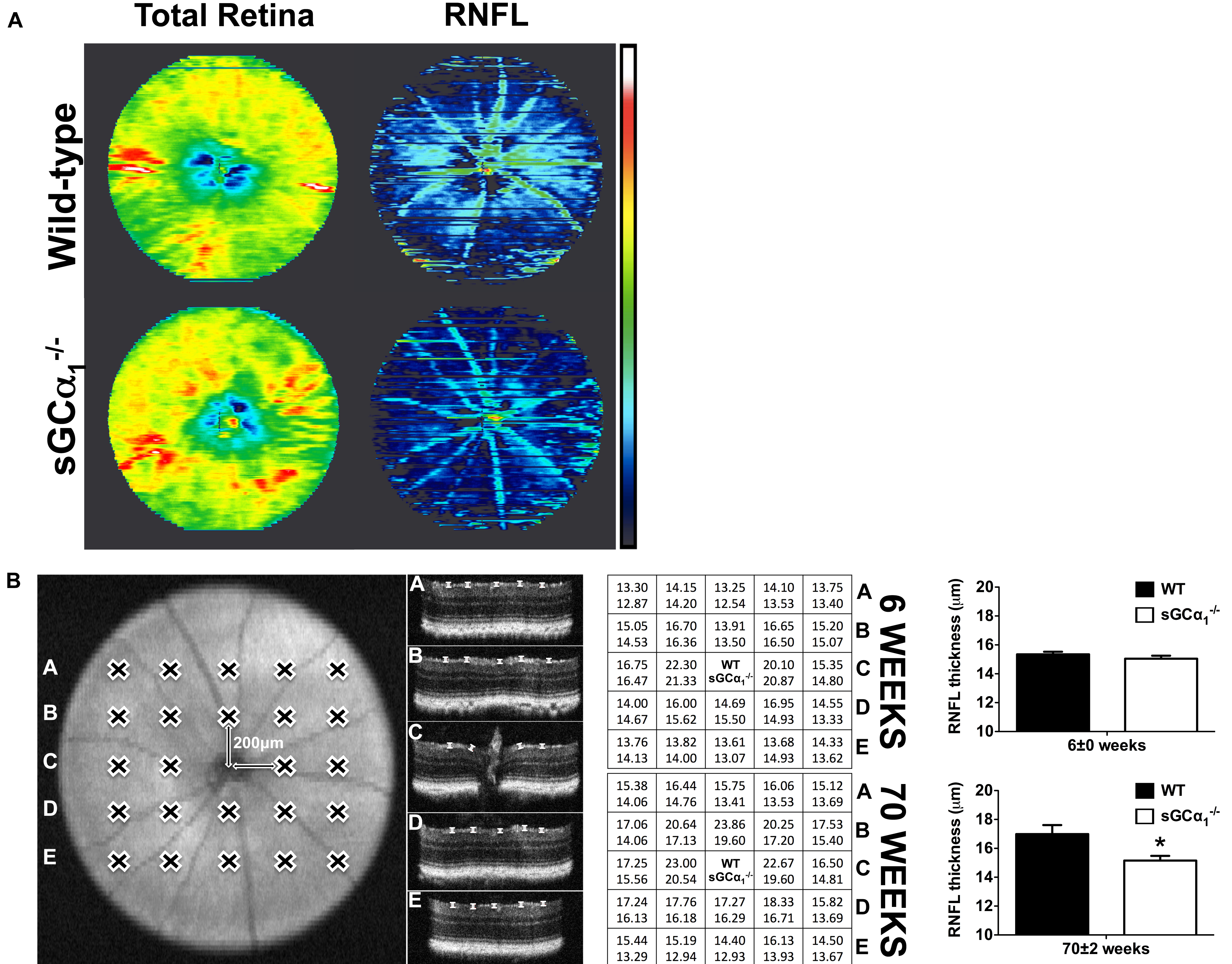

Supplement: Figure S1 — SD-OCT analysis of retinal nerve fiber layer thickness in sGCα1−/− mice. A: Representative heat maps of absolute total retinal thickness and RNFL thickness in 12 month-old wild-type (WT) and soluble guanylate cyclase α1-deficient (sGCα1 −/−) mice. No differences in total retinal thickness were detected between sGCα1 −/− and WT mice (fig. 2A). RNFL thickness was thinner in sGCα1 −/− mice than in WT mice, as demonstrated by the darker blue colors in the sGCα1 −/− RNFL heat map (see also fig. 2A). Color scale varies between 1.53 (black) and 247.93 (white) μm. See fig. 2A for quantitative data. B: Validation of the automated segmentation analysis. The manually determined RNFL thickness data recapitulated the automated segmentation analysis and confirmed that the RNFL is thinner in old in sGCα1 −/− mice than in old WT mice. Left panel: Representative en-face image of the retina acquired using SD-OCT imaging (left), showing the 24 points (marked by X) of a 5×5 grid at which RNFL thickness was measured, and B-scans (right, A-E) showing placement of the 24 calipers used to measure RNFL thickness. Middle panel: mean RNFL thickness measured in all 24 pre-defined points in WT (upper number) and sGCα1 −/− mice (lower number). Right panel: mean RNFL thickness (as averages of RNFL thickness measured manually in 24 points), assessed using a 2-way repeated measures ANOVA, did not differ in 6-week-old sGCα1 −/− and age-matched WT mice (n = 15 and 20, respectively; P = 0.29), but was thinner in 70-week-old sGCα1 −/− mice than in age-matched WT mice (n = 17 each; *P = 1.4×10^−2^). (TIF) [file pone.0060156.s001.tif]

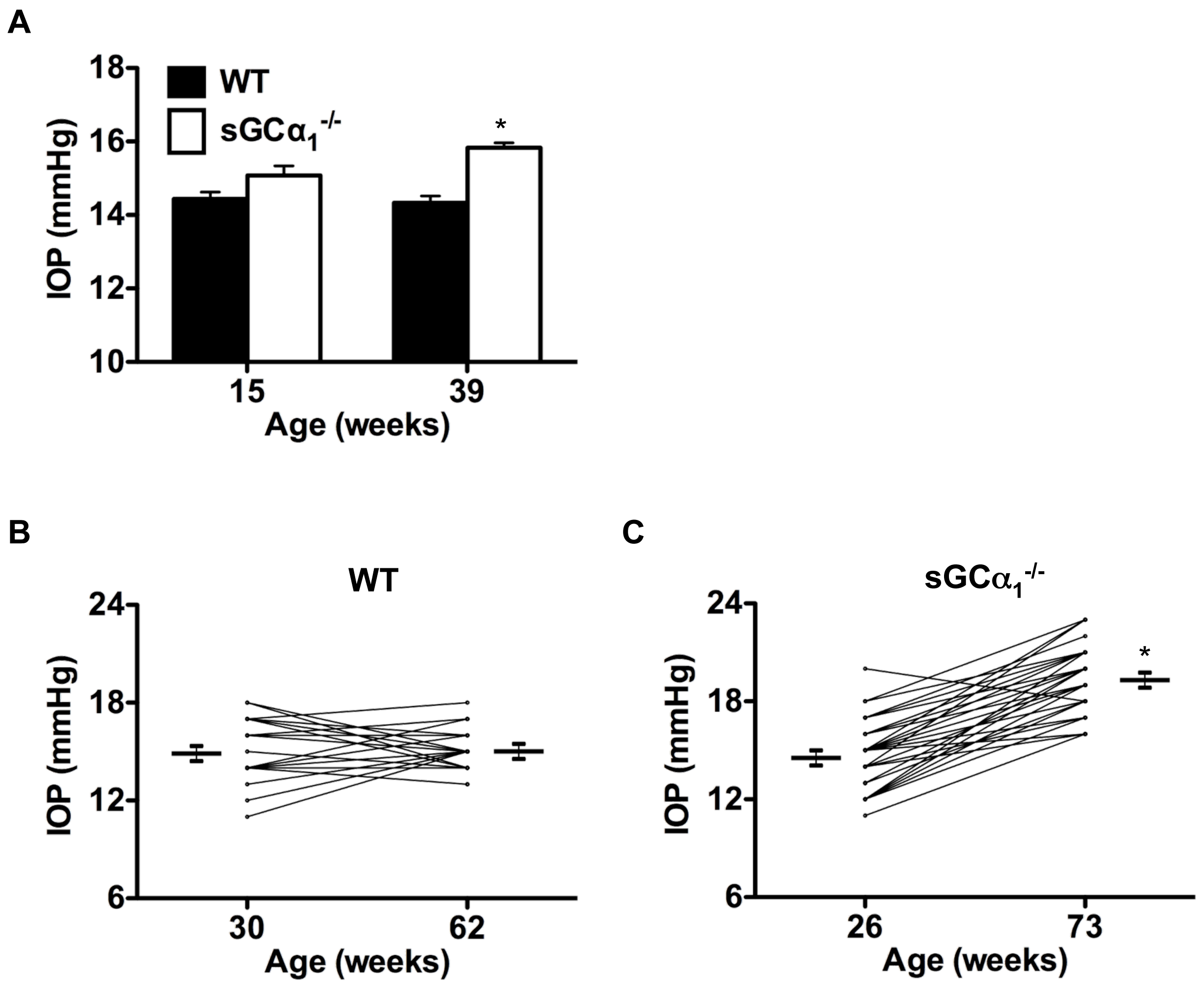

Supplement: Figure S2 — Intraocular pressure (IOP) increases with age in sGCα1−/− mice. A: IOP in young (15±6-week-old) and old (39±14-week-old) wild-type (WT) and soluble guanylate cyclase α1-deficient (sGCα1 −/−) mice. No statistically significant difference in IOP was detected between young sGCα1 −/− and age-matched WT mice (n = 61 and 112, respectively; P = 0.08). In contrast, IOP was higher in old sGCα1 −/− mice than in age-matched WT mice (n = 468 and 140, respectively). *P = 2.9×10^−7^ vs. 39±14-week-old WT. B: IOP in a second independent cohort of WT eyes (n = 27) measured serially at 2 time points. IOP was 15±2 and 15±1 mmHg in 30±0 and 62±0-week-old WT mice, respectively. P = 0.93. C: IOP in a second independent cohort of sGCα1 −/− eyes (n = 41) measured serially at 2 time points. IOP increased from 15±2 to 19±2 mmHg in 26±4 and 73±5-week-old sGCα1 −/− mice, respectively. *P = 4.8×10^−16^. (TIF) [file pone.0060156.s002.tif]

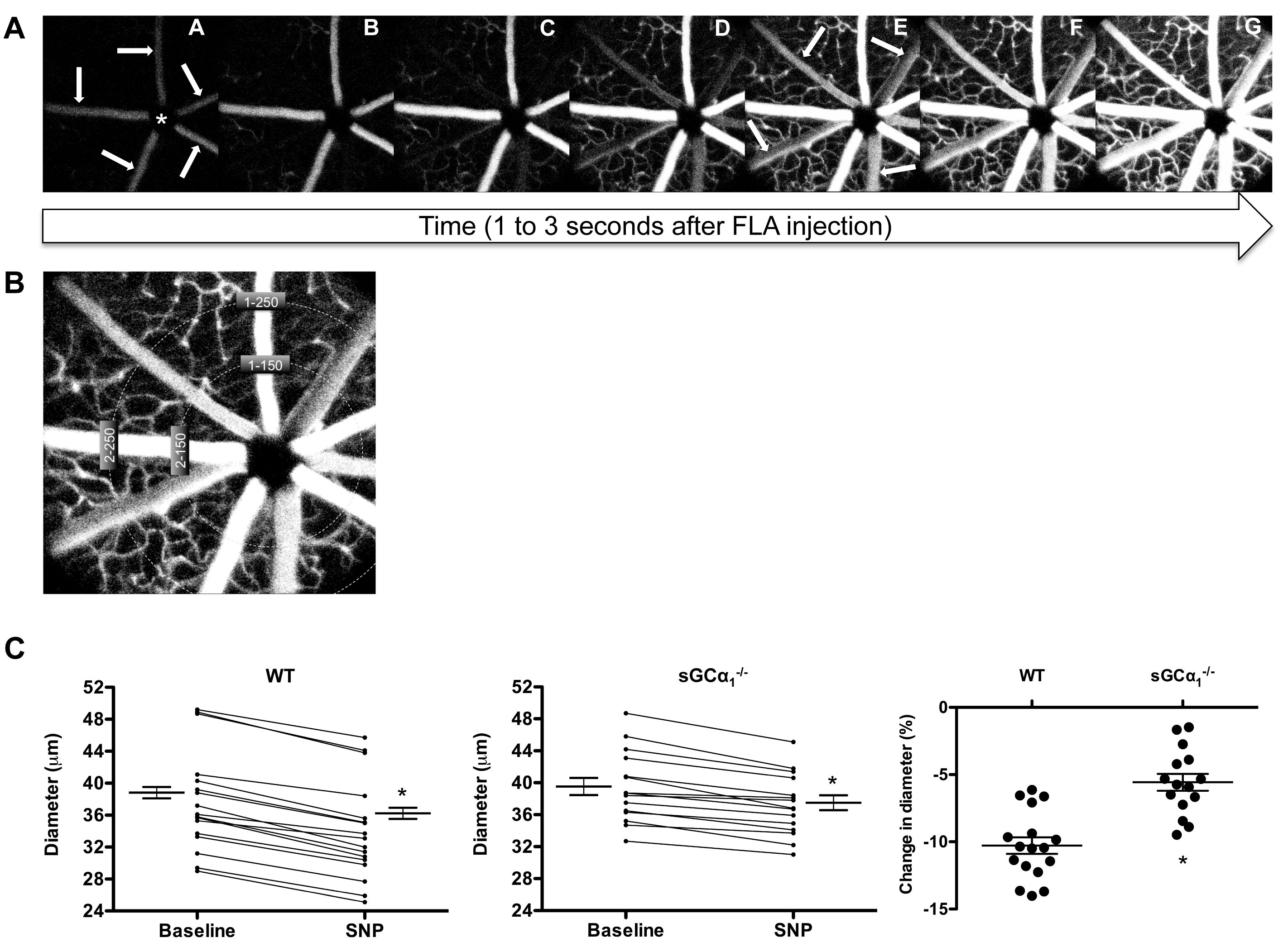

Supplement: Figure S3 — Fluorescein angiography in WT and sGCα1−/− mice. A: Serial images (A–G) of fluorescein angiography between 1 and 3 seconds after I.V. injection of fluorescein. Retinal arterioles (arrows in panel A) were identified based on earlier filling with fluorescein than veins (arrows in panel E). B: Image of fluorescein angiography. The width of the arterioles was determined (boxes) at fixed distances from the optic nerve head (inner dashed ring = 150 µm; outer dashed ring = 250 µm). C: Quantitative analysis of the diameter of retinal arterioles in wild type (WT, left panel) and soluble guanylate cyclase α1-deficient mice (sGCα1 −/−, middle panel) before and after a challenge with 0.8 mg/kg sodium nitroprusside. Baseline diameter of retinal arterioles was similar in WT and sGCα1 −/− mice (P = 0.40). Injection of 0.8 mg/kg sodium nitroprusside reduced the diameter of retinal arterioles in both WT (*P = 7.8×10^−12^) and sGCα1 −/− mice (*P = 1.7×10^−6^). The change in diameter induced by injection of sodium nitroprusside was more pronounced in WT than in sGCα1 −/− mice (right panel, *P = 9.0×10^−6^). n = 17 and 15 WT and sGCα1 −/− arterioles, respectively; 3–4 arterioles/mouse from 5 mice, each. See also fig. 6B for data analysis per mouse. (TIF) [file pone.0060156.s003.tif]
